# Giant pedunculated hepatocellular carcinoma with hemangioma mimicking intestinal obstruction

**DOI:** 10.1186/1471-230X-11-99

**Published:** 2011-09-22

**Authors:** Theodore Karatzas, Anastasios Smirnis, Dimitrios Dimitroulis, Dimitrios Patsouras, Kostantinos Evaggelou, Stylianos Kykalos, Gregory Kouraklis

**Affiliations:** 1 2nd Propedeutic Department of Surgery, "Laiko" General Hospital, 17 Ag. Thoma str, 11527 Goudi, University of Athens, Medical School, Athens, Greece; 2 Department of Pathology, University of Athens, Medical School, Mikras Asias 75, 11527, Goudi Athens, Greece

## Abstract

**Background:**

Pedunculated hepatocellular carcinoma (P-HCC) has rarely been reported and is characteristically large and encapsulated. Only sporadic cases have been published, in which P-HCC was combined with other liver tumors (mostly benign), making the diagnosis difficult.

**Case presentation:**

We report a patient who was admitted to our hospital with clinical features of intestinal obstruction and a palpable mass in the right iliac fossa. Ultrasound, computed tomography and magnetic resonance imaging demonstrated an encapsulated mass of unclear origin and characteristics of liver hemangioma. Laboratory tests revealed elevated α-fetoprotein (> 800 ng/ml) and cancer antigen 125 (> 51.2 U/ml). With a possible diagnosis of giant liver hemangioma, we proceeded to surgery. During surgery, a giant pedunculated tumor was discovered on the inferior surface of the right lobe of the liver, hanging free in the right abdominal cavity towards the right iliac fossa. The macroscopic appearance of the tumor was compatible with liver hemangioma. Tumor resection was performed at a safe distance, including the pedicle. The rest of the liver appeared normal. Histopathological examination revealed grade II and III HCC (according to Edmondson-Steiner's classification) with nodular configuration, central necrosis, and infiltration of the capsule. Underneath the tumor capsule, residual tissue of a cavernous hemangioma was recognized. The resection margins were free of neoplastic tissue.

**Conclusion:**

This rare presentation of a giant P-HCC combined with a hemangioma with features of intestinal obstruction confirmed the diagnostic difficulties of similar cases, and required prompt surgical treatment. Therefore, patients benefit from surgical resection because both the capsule and the pedicle prevent vascular invasion, therefore improving prognosis.

## Background

Pedunculated hepatocellular carcinoma (P-HCC) is a rare form of cancer, which protrudes from the liver as a massive tumor with or without a pedicle. The exophytic growth of the tumor lying beyond the confines of the liver occasionally poses a considerable diagnostic challenge. This is due to the uncertainty of the origin of the tumor and that it mimics other abdominal tumors. An incidence of P-HCC of 0.24-3.0% has been reported in Japan [1].

Pedunculated HCC variants are classified as nodular, diffuse and massive [2,3]. The mechanism for extrahepatic growth remains unknown. Most investigators support that tumor size plays a role in the prognosis and significantly affects survival [4,5]. Furthermore, large tumors are associated with a significantly higher risk of recurrence [2,5]. The spatial relationship between the liver and an extrahepatically pedunculated growing mass varies considerably between cases. In this report, we describe a case of giant P-HCC combined with hemangioma, in the right iliac fossa, which presented with clinical features of intestinal obstruction.

## Case presentation

**Figure 1:**
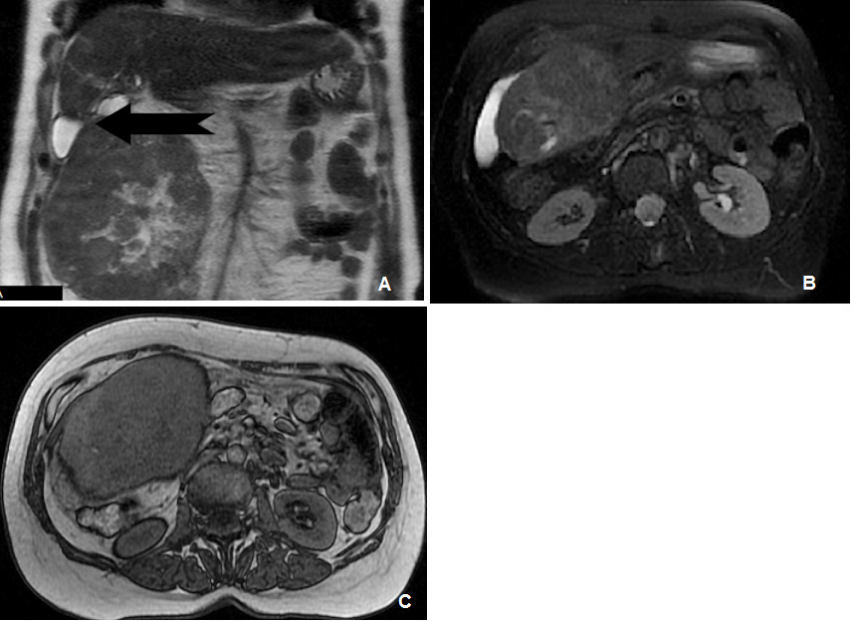
**MRI images of the pedunculated tumor**. **A: **MR T2 coronal image showing a heterogeneous tumor mass underneath the right hepatic lobe. Arrow indicates tumor pedicle. **B: **MR T2 fat suppressed axial image depicting a heterogeneous signal intensity liver mass. **C: **MR T1 axial image showing a large right liver lobe mass displacing intestinal loops.

A 63-year-old woman with a history of idiopathic hemochromatosis and treatment with phlebotomies was admitted to our emergency department with a 2-day history of right abdominal pain, which she described as colicky, and clinical characteristics of intestinal obstruction. She complained of acute retention of gas and flatus and she had no bowel movements in the previous 3 days. She also complained of nausea, vomiting and no food intake. The patient reported anorexia, severe weakness and weight loss of 12 kg over a period of 6 months. On physical examination, her abdomen was markedly tender at the right iliac fossa and a vague mass was palpated. Her bowel sounds were of obstructive type. The biochemical evaluation tests were within normal limits. The most important laboratory findings were elevated α-fetoprotein (> 800 ng/ml) and cancer antigen 125 (51.2 U/ml). HBV and HCV serum blood tests investigated in the past were negative. Abdominal X-ray showed opacity in the right upper abdomen, displacing the small bowel loops, and a few radiological signs of intestinal obstruction. The patient had clinically abdominal distention and features of intestinal obstruction on physical examination therefore, ultrasound was not performed. She also reported a history of liver hemangioma that was diagnosed 10 years previously. Following computed tomography (CT) and magnetic resonance imaging (MRI), without administration of intravenous contrast agent, a large (14 × 12.5 cm) well-defined, heterogeneous mass of unclear origin and nature was detected between the right hepatic lobe and the colon. The mass appeared hyper-dense on T_1_- and T_2_- weighted images compared with the surrounding lower-signal-intensity liver tissue. No invasion into the liver or adjacent organs was demonstrated (Figure 1).

**Figure 2:**
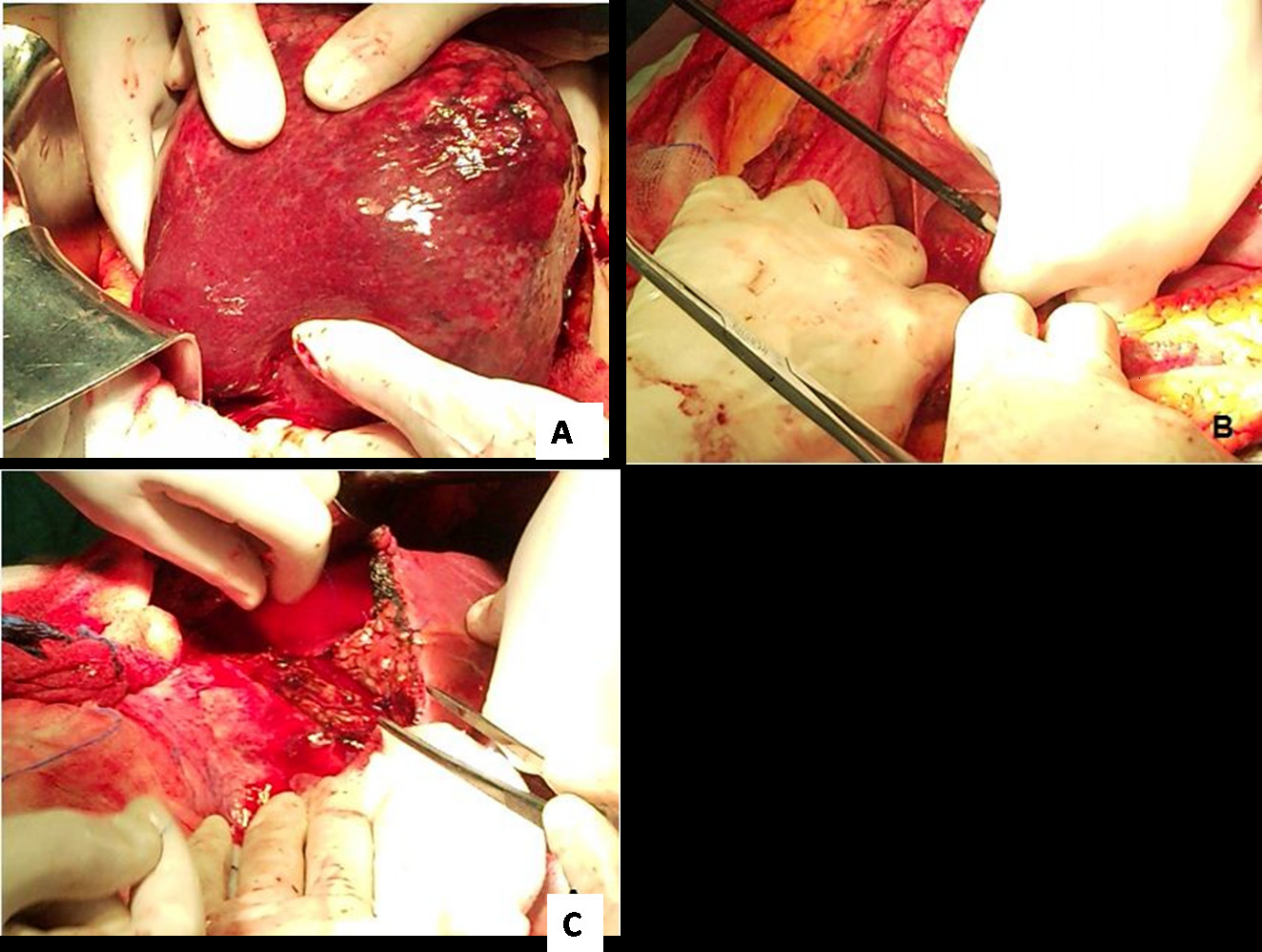
**Intraoperative sequence of the pedunculated HCC resection (A, B, C)**.

With a possible diagnosis of giant liver hemangioma causing acute abdominal discomfort and intestinal obstruction, surgery followed. During the operation, an enormous pedunculated tumor with exophytic growth and a thin pedicle attached only to the inferior surface of the right lobe of the liver was found, hanging freely in the right abdominal cavity (Figure 2).

**Figure 3:**
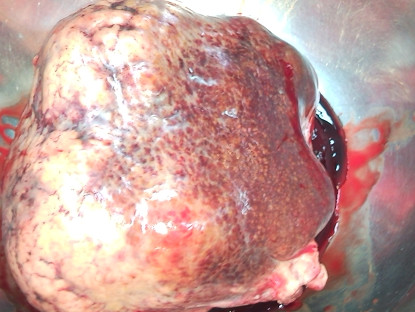
**Resected tumor mass**.

**Figure 4:**
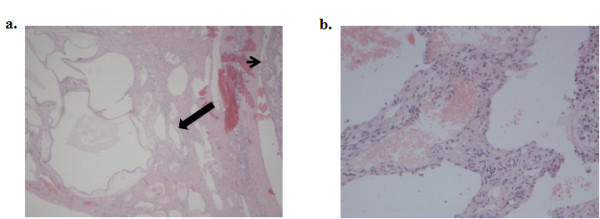
**Histological images**. **A**. Histological section (H&E stain) of the described cavernous hemangioma (arrow) located underneath the hepatic capsule, adjacent to the HCC (arrowhead) (magnification × 100). **B**. Areas of hemangioma at higher magnification(× 200).

The remainder of the liver was of normal color and consistency. Grossly, the tumor was fairly well-circumscribed and encapsulated, invading veins in the periphery. This gave the impression of hemangioma (Figure 3). No enlarged porto-hepatic lymph nodes were seen or palpated. The patient underwent resection of the tumor from its pedicle close to the liver edge, at a safe distance from the tumor mass. The postoperative course of the patient was uneventful. Histopathological examination revealed a white-grayish tumor weighing 920 g and measuring 14 × 12.5 × 9.2 cm with nodular configuration and central necrosis (Figure 4). Microscopically, the tumor exhibited the morphological features of grade II and III HCC, according to Edmondson-Steiner's classification. There was invasion of a few small veins and of the hepatic capsule, without it being disrupted. Scarring, broad fibrous septa and extensive central necrosis were also observed. Adjacent to the tumor and underneath the capsule, residual tissue of a cavernous hemangioma with a small thrombus was recognized. The resection margins were free of neoplastic tissue. Liver parenchyma from the resection margin of tumor pedicle exhibited features of mild reactive non-specific hepatitis, signs of portal venous stasis and mild microvesicular steatosis. No cirrhosis or iron deposition signs were detected.

Follow-up of the patient has been short, but 5 months after surgery there was no evidence of recurrent disease.

## Discussion

HCC occasionally grows outside the liver in a pedunculated form, hence making preoperative diagnosis difficult [1,2]. To differentiate P-HCC from other exophytic benign and malignant liver tumors is not always absolute, particularly when other pathological hepatic lesions coexist [6-9]. Benign pedunculated tumors such as focal nodular hyperplasia, liver cell adenoma, hemangioma and angiomyolipoma often pose a diagnostic challenge due to uncertainty of the nature of the tumor [7]. As for malignant tumors, the differential diagnosis is occasionally difficult in cases of exophytic growth of an HCC protruding caudad from the right lobe of the liver, which may show retroperitoneal extension, thus mimicking a right adrenal tumor [10,11]. Similarly, pedunculated HCC may invade the duodenum and mimic a duodenal gastrointestinal stromal tumor [6]. Although the use of various imaging modalities such as CT and MRI can demonstrate the presence of the tumor itself, making a correct diagnosis is often challenging for radiologists because of the uncertainty of tumor origin [6,12]. The characterization of the nature of these tumors is of primary importance for their management and treatment. Specific imaging features, such as "prominent feeding artery sign", dynamic diffuse enhancement pattern during hepatic arterial phase, and fatty components are useful for identifying the origin and nature of liver tumors [6,8,9].

In the present case, the patient was aware of a liver mass of unknown nature, which was asymptomatic for over 10 years. Due to idiopathic hemochromatosis, the patient had regular follow-up investigations with ultrasound and CT, suggesting an exophytic mass arising from the inferior surface of the right lobe of the liver, and with imaging features characteristic of hemangioma. It is reported that Idiopathic hemocromatosis is associated with an increased risk for hepatocellular carcinoma [13,14]. The risk has been estimated to be as high as 200-fold increased and occurs predominantly in patients with cirrhosis at the time of diagnosis. Even if excess iron has been removed by phlebotomies, the risk of HCC persists once cirrhosis has been established. Iron overload per se might also contribute to the development of HCC. In the case of our patient, the liver parenchyma from the surgical margin of tumor pedicle exhibited features of mild reactive non-specific hepatitis, signs of portal venous stasis and mild microvesicular steatosis. No cirrhosis or iron deposition signs were present. Furthermore, iron has successfully been depleted by phlebotomies, so excessive iron deposition could not be detected in liver parenchyma by histology examination. Other cofactors that might promote the development of HCC in hemochromatosis, such as viral hepatitis, excessive alcohol intake etc. were also absent. In literature, do not exist reports on a potential association between the specific subtype of P-HCC and idiopathic hemochromatosis.

This patient unfortunately experienced a severe allergic reaction to radio-opaque medium during CT examination in the past and all CT and MRI studies were performed without administration of contrast medium. Therefore, typical enhancement patterns of the exophytic HCC could not be identified. It is well known that, most hepatic hemangiomas identified on CT or MRI can be diagnosed accurately from the characteristic imaging features of these lesions. On T1 weighted (T1W) images are hypointense to surrounding hepatic parenchyma with smooth, well-defined, often lobulated margins. On T2 weighted images (T2 W) they become significantly hyperintense compared to normal liver. In this case of a giant pedunculated HCC with hemangioma the MRI was not characteristic. According to Bader TR et al. [7], the exophytic benign tumors of the liver, on preconstant images, signal intensity was regarded as isointense, hypointense, or hyperintense in comparison to normal liver parenchyma. However, in our case, the heterogeneous nature of tumor mass resulted in a different imaging appearance on MRI.

The patient's condition had shown a gradual deterioration in the previous 6 months, accompanied by general malaise, anorexia, severe weight loss, and persistent right hypochondrial pain. At admission, MRI examination showed an increased size of the pedunculated liver mass that appeared hyper-dense on T_1_-and T_2_- weighted images, with the central portion of the tumor showing a high signal intensity area compatible with central necrosis. Although the imaging studies could not draw a definitive diagnosis of the nature of the liver tumor, we proceeded to emergency surgery based on the patient's clinical picture and the findings of a palpable abdominal mass. The exophytic growth of P-HCC usually tends to form a mass that expands into the surrounding organs, rather than infiltrating them.

The histological findings of this patient were very unusual, since P-HCCs are rare liver tumors and to our best knowledge there have been no reports in literature describing P-HCC combination to other tumors. The largest series dealing with the surgical results of pedunculated HCCs is reported by Yeh CN et al. [2], where no combination with other tumors has been observed.

According to their macroscopic appearance, P-HCCs are classified into pedunculated type with a pedicle (sub-type I, as is this case) and pedunculated type without a pedicle and attached to the liver surface (sub-type II) [3]. Horie et al. [5] have reported that almost all P-HCCs show poorly differentiated characteristics, using Edmondson and Steiner's classification [15]. Yeh et al. [2], in a large study of 18 hepatic pedunculated tumors from 432 patients with HCC, found that the P-HCC group with larger tumors (> 5 cm) had a significantly favorable overall survival (94 months), compared to lower survival (54.5 months) of the non P-HCC type II group. The pedunculated tumors tended to be larger and to have a more prominent capsule. Although increased tumor size is associated with poorer prognosis in HCC due to increased invasiveness, the patients with pedunculated tumors and pedicle have larger tumors but do not have a significantly decreased survival. Pedunculated tumors exhibit less vascular invasion than do non P-HCCs. Vascular invasion is widely accepted as the most consistently reported risk factor for recurrence after resection, which explains the improved survival seen in the P-HCC group of patients [2,5,16]. In our case, the presence of a long pedicle without vascular invasion made resection of the tumor easily controllable and allowed for gross disease clearance, which hopefully will lead to better prognosis and long-term survival.

## Conclusions

In cases of pedunculated hepatic tumors, correct diagnosis is difficult to obtain. Nevertheless, patients benefit from surgical resection, especially in cases with large P-HCC without vascular invasion.

## Consent

Written informed consent was obtained from the patient for publication of this case report and any accompanying images. A copy of the written consent is available for review by the Editor-in-Chief of this journal.

## Competing interests

The authors declare that they have no competing interests

## Authors' contributions

TK and DD performed the surgery and were involved in drafting the manuscript; AS, DP and SK organized the patient's data and figures and helped to draft the manuscript; KE carried out the histopathology examination; GK corrected and contributed to the final version of the manuscript; TK wrote the manuscript. All authors read and approved the final manuscript.

## Pre-publication history

The pre-publication history for this paper can be accessed here:

http://www.biomedcentral.com/1471-230X/11/99/prepub
